# Gram-negative bacterial bloodstream infections in cancer patients: a study of epidemiological trends and antibiotic susceptibility

**DOI:** 10.1186/s41182-025-00811-8

**Published:** 2025-10-07

**Authors:** Bircan Kayaaslan, Ayşe Kaya Kalem, Fatıma Bolat, Tugba Cınar, Gulen Dönertas Turan, Sezgin Pepeler, Tugba Kos, Bedia Dinc, Rahmet Guner

**Affiliations:** 1 Department of Infectious Disease and Clinical Microbiology, Ankara Yildirim Beyazit University, Ankara Bilkent City Hospital, Bilkent Street No:1, 06800 Ankara, Turkey; 2 Department of Infectious Disease and Clinical Microbiology, Ankara Bilkent City Hospital, Ankara, Turkey; 3 Department of Hematology, Ankara Bilkent City Hospital, Ankara, Turkey; 4 Department of Medical Oncology, Ankara Bilkent City Hospital, Ankara, Turkey; 5 Department of Microbiology, University of Health Sciences, Ankara Bilkent City Hospital, Ankara, Turkey

**Keywords:** Bacteremia, Gram-negative bacterial infections, Drug resistance, bacterial, Microbial sensitivity tests, Neoplasms

## Abstract

**Purpose:**

The primary aim of this study was to determine the epidemiological characteristics of Gram-negative bloodstream infections (BSIs) and the susceptibility profiles of the causative microorganisms in hospitalized patients with hematological and oncological malignancies.

**Methods:**

This retrospective observational study investigated Gram-negative BSIs in patients over the age of 18 with a diagnosis of malignancy admitted to the hematology and oncology departments of Ankara Bilkent City Hospital between June 1, 2019, and September 1, 2023. Demographic data, disease activation status (new diagnosis, remission and relapse/refractory disease), causative Gram-negative microorganisms, and antibiotic susceptibilities were recorded.

**Results:**

A total of 435 patients with 569 Gram-negative bacteremia were included. The median age of the patients was 56 years (range: 18–90 years), and 60.7% were male. Among the BSI episodes, 11.1% were catheter related. The most common Gram-negative pathogens were *E. coli* (40.6%), *Klebsiella* spp. (28.8%), and *Pseudomonas* spp. (9.1%). Of all isolates, 47.7% were multidrug-resistant (MDR), and 26.7% produced extended-spectrum beta-lactamase (ESBL). The rates of ESBL resistance were 40.3% and 28.7% for *E. coli* and *Klebsiella* spp., respectively, whereas the carbapenem resistance rates were 9.1% and 27.4%, respectively. Meropenem susceptibility rates were 93.4% for *E. coli*, 79.1% for *Klebsiella* spp., and 72.9% for *Pseudomonas* spp. MDR was most frequent in *Acinetobacte*r spp. (56.5%) and *Klebsiella* spp. (56.1%), with the highest carbapenem resistance in *Acinetobacter* spp. (52.2%).

**Conclusion:**

This study highlights the high prevalence of MDR Gram-negative pathogens, limiting options for effective empirical therapy. The elevated rates of ESBL production, and carbapenem resistance are concerning and underscore the need for strengthened antimicrobial stewardship, updated guidelines, and new treatment options for managing Gram-negative BSIs.

## Introduction

Patients with hematological and oncological malignancies are vulnerable to infections during the period of febrile neutropenia resulting from three issues: the disease itself, from chemotherapy, or after hematopoietic stem cell transplantation (HSCT). Bloodstream infections (BSIs) are the most common serious infectious complications and account for approximately 30% of all episodes of febrile neutropenia in adult patients with malignancies [1, 2]. The reported crude mortality rates of BSIs in cancer patients are as high as 42%, and the attributed mortality rate is as high as 30% [3].

The bacterial etiology and antimicrobial resistance rates have changed over time as a consequence of the increased use of quinolone prophylaxis, frequent hospitalizations, the use of broad-spectrum antibiotics, and invasive procedures [1, 4–6]. In recent years, a significant shift in the etiology of BSIs from Gram-positive to Gram-negative organisms and increased multidrug resistance have been reported in cancer patients [4–7]. Patients with BSIs due to multidrug resistant (MDR) pathogens have a higher percentage of inappropriate initial antibiotic therapy than those with BSIs caused by non-MDR pathogens [3]. Inappropriateness of empirical antibiotic therapy has been reported to be associated with increased mortality [8–12]. Knowing the antimicrobial resistance patterns of common Gram-negative pathogens in patients with suspected BSIs and guiding appropriate empirical antibiotic therapy is crucial in optimizing treatment and reducing mortality in sensitive populations such as the patient group with BSIs. [1, 8]. Bacterial dominance and multidrug resistance are dynamic processes that may differ across different geographic locations with different antibiotic use preferences or habits, and each center's comprehensive knowledge of its patient population will help guide appropriate empirical antibiotic selection. In this study, we aimed to analyse the Gram-negative bacterial etiology and antimicrobial resistance trends of BSIs in hemato-oncological patients at quaternary public hospitals.

## Material methods

### Study design and patient population

All patients older than 18 years who were diagnosed with Gram-negative BSIs via hematology, bone marrow transplantation, or oncology units at Ankara Bilkent City Hospital, a quaternary care hospital, between June 1, 2019 and September 1, 2023 were included in the study. In these units of the hospital, there are a total of 136 beds, 48 in hematology units, 16 in bone marrow transplantation units and 72 in oncology units. All patients were included in the study, regardless of whether they had neutropenia. Ethical approval was obtained from the Ankara City Hospital Ethical Committee 1 (No: E1-23-4001). All adult patients with malignancy who were hospitalized in the hematology and oncology clinic and had a Gram-negative BSI were included in the study regardless of the source of infection. All patients, both neutropenic and non-neutropenic, regardless of whether they received chemotherapy and whether the infection developed inside or outside the hospital, were included in the study. All hematological malignancies (e.g., leukemias, lymphomas, myelodysplastic syndromes, multiple myeloma, and other hematologic cancers) and all solid organ malignancies were included in the study. Patients followed for diagnoses other than malignancy and those with incomplete information in their records were excluded from the study. Demographic and laboratory data were retrospectively screened via the hospital automation database system.

### Definitions and classification

Malignancies were categorized into three groups on the basis of their disease activity: new diagnosis, remission, and relapse/refractory disease. Patients with complete and partial responses were included in the remission group, and those with progressive/resistant or recurrent disease were included in the relapse-refractory class.

Bacteremia was categorized on the basis of its source into catheter-related and noncatheter-related BSIs. In accordance with the Infectious Diseases Society of America’s (IDSA) guidelines for catheter-associated infection, differential time to positivity was used for the diagnosis of catheter-related BSI (CR-BSI); the growth of the same microorganism in blood cultures was taken simultaneously from the central venous catheter and peripheral vein, and the detection of the microorganism in the culture was taken from the catheter lumen 2 h or earlier than that in the peripheral blood. Since quantitative culture could not be performed in our laboratory, the growth amount was not used for the diagnosis of CR-BSI [13]. All bacteremia that did not meet the definition of CR-BSI was classified as noncatheter-related BSI, and the source of bacteremia was not recorded.

### Microbiological examination

In routine practice, at least one set of blood cultures consisting of two bottles, one anaerobic and one aerobic, is taken from each patient who becomes febrile in hemato-oncology clinics. The growth of at least one Gram-negative microorganism in the blood culture was considered a BSI. Repetitive growth, fungal growth, growth of microorganisms thought to be contaminated, or growth of more than two microorganisms in blood cultures were not included. More than one episode in a patient and the growth of two different Gram-negative were included in the study.

A BactAlert (bioMerieux/French) automated blood culture system was used for monitoring blood culture bottles. Gram-negative were identified at the species level via the VitekMS (bioMerieux/French) device and the MALDI-TOF MS method (bioMerieux, Lyon, France). Antimicrobial susceptibility tests were performed with a Vitek2 (bioMerieux/France) automated system. Colistin susceptibility was determined using the broth microdilution method in accordance with ISO 20776-1, employing detergent-free, cation-adjusted Mueller–Hinton broth [14]. For ceftazidime–avibactam, the EUCAST disk diffusion method was applied using 10/4 µg disks [15]. The results of the antimicrobial susceptibility tests were evaluated and interpreted in accordance with the criteria of the European Committee on Antimicrobial Susceptibility Testing (EUCAST) for each year [16].

All data on antibiotic susceptibility in Gram-negative bacteria were recorded. Analyses of susceptibility rates were done only for strains for which susceptibility data were reported. In the present study, this encompassed the analysis of susceptibility rates to antibiotics for *the Escherichia* coli, *Klebsiella* spp., *Enterobacter* spp., *Pseudomonas* spp., *Acinetobacter* spp., and *Stenotrophomonas maltophila* strains. MDR bacteria were defined as nonsusceptible in cases where they were resistant to at least one agent in three or more classes of antimicrobials in accordance with the international consensus proposed by Magiorakos et al*.* Extended-spectrum β-lactamases (ESBLs) are plasmid- or chromosome-encoded enzymes that confer resistance to extended-spectrum cephalosporins (e.g., cefotaxime, ceftazidime) and monobactams (e.g., aztreonam) through hydrolysis, while remaining susceptible to inhibition by betalactamase inhibitors such as clavulanic acid [17]. The automated VITEK system (bioMérieux, France) aids in the identification of ESBL-producing *Enterobacterales*. Accordingly, ESBL production alone was not considered sufficient for MDR classification unless the overall resistance phenotype met the ≥ 3 antimicrobial category criterion. Likewise, microorganisms with intrinsic resistance mechanisms, such as *Stenotrophomonas maltophilia*, were not automatically categorized as MDR; these were reported separately as intrinsically resistant organisms, unless they additionally fulfilled the MDR definition [18].

### Statistical analysis

Continuous variables were summarized as mean ± standard deviation when approximately normally distributed and as median (interquartile range) otherwise; categorical variables were presented as numbers (percentage). Distributional assumptions were evaluated using the Shapiro–Wilk test with visual inspection of histograms and Q–Q plots. Between-group comparisons used the independent-samples t test or Mann–Whitney U test for two groups; categorical variables were compared using the χ^2^ test or Fisher’s exact test as appropriate. All tests were two-sided, and a *p* value < 0.05 was considered statistically significant. Analyses were performed with IBM SPSS Statistics, version 21.

## Results

### Study population

From 1 June 2019 to 1 September 2023, a total of 435 cancer patients who had at least one Gram-negative bacteremia episode were included in the study. Among the patients, 60.7% were male, and the median age was 56 years (min–max 18–90). Among the episodes, 67.3% occurred in patients with a new diagnosis, 20% in those with the disease in remission, and 15.7% in those with relapsing-refractory disease. In 11.1% of the episodes, the source of Gram-negative bacteremia was the catheter (Table 1).

**Table 1:** Demographic profile and characteristics of patient with Gram-negative bacteriemia on hemato-oncological patients

Characteristics	*n* (%)
Age, median years (min–max) (*n* = 435)	56 (18–90)
Gender, (*n* = 435)	
Male	264 (60.7)
Female	171 (39.3)
Clinic of patients during bacteremia (*n* = 569)	
Hematology	293 (51.5)
Medical oncology	276 (48.5)
Hospitalization year (*n* = 569)	
2019*	67 (11.8)
2020	123 (21.6)
2021	112 (19.6)
2022	163 (28.6)
2023**	104 (18.2)
Disease status (*n* = 569)	
New diagnosis	383 (67.3)
Remission	114 (20.0)
Relapse/refractory	72 (12.7)
Source of Gram-negative bacteria (*n* = 569)	
Catheter related	63 (11.1)
Non-catheter related	506 (88.9)
The antibiotic resistance profile of Gram-negative bacteria (*n* = 569)	
MDR	272 (47.7)
ESBL	152 (26.7)
Carbapenem resistant	74 (13.0)

Data are presented as n (%) unless noted otherwise. Demographical parameters (age and gender) are analyzed for a total 435 patients, other parameters (clinic where the patient is admitted, hospitalization year, disease status, source of bacteremia, resistance prophile) are analyzed for a total 569 Gram-negative bacteremia episodes

MDR: Multidrug resistant; ESBL: Extended-spectrum beta-lactamases

^*^ Only patients from 1 June to 31 December (for six months)

^**^Up to September 1, 2023 (for eight months)

### Microbiological examination

A total of 569 Gram-negative isolates were detected in hemato-oncological patients during the study period. Among the 569 bacteremia episodes, 293 (51.5%) occurred in patients with hematologic malignancies, and 276 (48.5%) occurred in patients with solid organ malignancies. The most common bacteriemic episodes (28.6%) were detected in the year 2022. The MDR, ESBL and carbapenem resistance rates in Gram-negative microorganisms obtained from BSI were 47.7%, 26.7%, and 13.0% respectively (Table 1).

The most common Gram-negative microorganisms identified during BSI episodes were *E. coli* (40.6%), *Klebsiella* spp. (28.8%), and *Pseudomonas* spp. (9.1%) (Table 2). The ESBL rates in the *E. coli* and *Klebsiella* spp. strains were 40.3% and 28.7%, respectively, and the carbapenem resistance rates were 9.1% and 27.4%, respectively. The highest MDR rates were detected in *Acinetobacter* spp. (56.5%) and *Klebsiella* spp. (56.1%) isolates, and the rates were similar. The highest carbapenem resistance rate (52.2%) was detected in *Acinetobacter* spp. isolates (Table 3).

**Table 2:** Distribution of Gram-negative bacteria from bloodstream infection in patients with hemato-oncological malignancy

Gram-negative bacteria isolated from blood culture	*n* (%)
*Escherichia coli*	231 (40.6)
*Klebsiella spp.*	164 (28.8)
*Pseudomonas spp.*	52 (9.1)
*Enterobacter spp.*	29 (5.1)
*Acinetobacter spp.*	23 (4.0)
*Stenotrophomonas maltophila*	14 (2.5)
*Serratia marcescens*	13 (2.3)
*Proteus mirabilis*	7 (1.2)
*Aeromonas spp.*	7 (1.2)
*Citrobacter spp.*	6 (1.1)
*Burkholderia cepacia*	5 (0.9)
Others	18 (3.2)

**Table 3:** ESBL, MDR and carbapenem resistance rates in Gram-negative bacteria obtained from bloodstream infections in patients with hemato-oncological malignancy

Resistance type	*Escherichia coli* (*n* = 231)	*Klebsiella* spp. (*n* = 164)	*Pseudomonas* spp. (*n* = 52)	*Enterobacter* spp. (*n* = 29)	*A. baumannii* (*n* = 23)
MDR	115 (49.8)	92 (56.1)	12 (23.1)	11 (37.9)	13 (56.5)
ESBL	91 (40.3)	47 (28.7)	–	7 (24.1)	–
Carbapenem resistant	21 (9.1)	45 (27.4)	13 (27.1)	3 (13.8)	12 (52.2)

MDR: Multidrug resistant, ESBL: Extended-spectrum beta-lactamases

The susceptibility to piperacillin-tazobactam, which is frequently used in clinical practice, was 67.7% in *E. coli*, 52.1% in *Klebsiella* spp. and 21.7% in *Pseudomonas* spp. isolates. Meropenem susceptibility was 93.4% in *E. coli*, 79.1% in *Klebsiella* spp. and 72.9% in *Pseudomonas* spp. isolates. Colistin susceptibility was between 83 and 97% among Gram-negative bacteria, and aminoglycoside susceptibility was between 52 and 93%. The levofloxacin and trimethoprim/sulfamethoxazole susceptibilities were 100% and 50%, respectively, in *S. maltophila* (Table 4).

**Table 4:** Antibiotic susceptibility rates of Gram-negative bacteria from bloodstream infections in patients with hemato-oncological malignancy

Antibiotics	*Escherichia coli* (*n* = 231)	*Klebsiella* spp. (*n* = 164)	*Pseudomonas* spp. (*n* = 52)	*Enterobacter* spp. (*n* = 29)	*A. baumannii* (*n* = 23)	*S. maltophila* (*n* = 14)
Penicillins						
Ampicillin	37/221 (16.7)	6/159 (3.8)	–	–	–	
Amoxicillin–clavulanate	73/223 (32.7)	60/157 (38.2)	–	0/28 (0)	–	
Piperacillin	–	–	5/36 (13.9)	–	–	
Piperacillin-tazobactam	155/229 (67.7)	85/163 (52.1)	10/46 (21.7)	16/25 (64.0)	6/18 (33.3)	
Cephalosporins						
Cefazolin	48/186 (25.8)	33/130 (25.4)	–	2/15 (13.3)	–	
Cefoxitin	140/204 (68.6)	91/149 (61.1)	–	1/27 (3.7)	–	
Cefotaxime	1/2 (50.0)	1/3 (33.3)	–	18/29 (62.1)	–	
Ceftriaxone	123/225 (54.7)	77/157 (49.0)	–	1/2 (50.0)	–	
Ceftazidime	123/229 (53.7)	70/164 (42.7)	20/51 (39.2)	17/28 (60.7)	7/18 (38.9)	2/4 (50.0)
Cefepime	127/221 (57.5)	75/157 (47.8)	13/50 (26.0)	16/25 (64.0)	–	
Ceftazidime-avibactam	7/7 (100)	19/23 (82.6)	5/5 (100)	2/3 (66.7)	–	
Quinolons						
Ciprofloxacin	102/228 (44.7)	85/164 (51.8)	10/49 (20.4)	20/28 (71.4)	0/18 (0)	
Levofloxacin	4/8 (50.0)	1/3 (33.3)	–	–	–	14/14 (100)
Carbapenems						
Meropenem	211/226 (93.4)	129/163 (79.1)	35/48 (72.9)	22/25 (88.0)	10/22 (45.5)	
Ertapenem	196/216 (90.7)	111/152 (73.0)	–	19/23 (82.6)	–	
Imipenem	122/128 (95.3)	67/89 (75.3)	–	16/18 (88.9)	9/20 (45.0)	
Aminoglycosides						
Amikacin	209/225 (92.9)	132/161 (82.0)	47/50 (94.0)	24/28 (85.7)	15/23 (65.2)	
Gentamicin	160/184 (87.0)	108/127 (85.0)	19/21 (90.6)	12/13 (92.3)	9/18 (50.0)	
Tobramycin	–	–	31/35 (88.6)	–	–	
Colistin	98/101 (97.0)	68/85 (86.1)	31/32 (96.9)	5/6 (83.3)	14/15 (93.3)	
Tigecycline	12/171 (5.2)	26/47 (55.3)	–	3/4 (75.0)	9/17 (52.9)	
TMP-SMZ*	93/226 (41.2)	87/160 (54.4)	–	20/28 (71.4)	9/19 (47.4)	8/14 (57.1)
Aztreonam	–	–	6/30 (20.0)		–	

Susceptibility rates were calculated only for strains for which susceptibility data were reported

^*^Trimethoprim/sulfamethoxazole

Hematological and solid organ malignancies were compared in terms of clinical and microbiologic parameters. Oncological patients with bacteremia had a statistically significantly higher age (0.005). No differences were found between the groups in terms of sex or MDR pathogen rates (respectively, 0.425 and 0462). The rate of two or more episodes was higher in patients with hematological malignancies than in those with solid organ malignancies (46.4% vs 33.0%, *p* 0.018). Catheters were the source of Gram-negative BSIs in 20.5% of hematological cancer patients, and this rate was significantly higher than the rate of 1.1% in oncological cancer patients (< 0.001). There was a difference in disease activity between the groups; 95.6% of the oncological cancer patients had new diagnoses, whereas 44.5% of the hematological cancer patients had new diagnoses (< 0.001) (Table 5).

**Table 5:** Comparison of clinical and microbiological parameters of Gram-negative bloodstream infections between patients with hematological and oncological malignancy

Characteristics	Hematological malignancy	Solid organ malignancy	*P* value
Age, median years (min–max) (*n* = 435)	50 (18–83)	60 (21–90)	0.005
Sex, male gender (*n* = 435)	121/ 211 (57.3)	143/224 (63.8)	0.425
Episode number (*n* = 569)			**0.018**
1 episode	157 (53.6)	185 (67.0)	
2 and more episodes	136 (46.4)	89 (33.0)	
Disease activity (*n* = 435)			** < 0.001**
New diagnosis	94 (44.5)	215 (95.6)	
Remission	76 (36.0)	3 (1.3)	
Relapse/refractory	41 (19.5)	7 (3.1)	
MDR rate	139 (47.4)	133 (48.2)	0.462
ESBL	70 (23.9)	82 (29.7)	
Carbapenem resistant	44 (15.0)	30 (10.9)	
Source of bloodstream infection (*n* = 569)			** < 0.001**
Catheter-related	60 (20.5)	3 (1.1)	
Non-catheter related	233 (79.5)	273 (98.9)	

Data are presented as n (%) unless noted otherwise. Demographical parameters (age, gender, disease activity) are analyzed for a total 435 patients; other parameters (episode number, source of bloodstream infection) are analyzed for a total 569 Gram-negative bacteremia episodes

## Discussion

BSIs are among the most important causes of increased mortality and morbidity in cancer patients. The proportion of Gram-negative bacteremia among all BSIs varies widely, ranging from 43.5% to 93.0% [1, 3, 5, 7, 19–22]. Much evidence from recent studies has shown that Gram-negative bacteria have reemerged as the predominant causative pathogens of BSI in cancer patients [20–24]. In this retrospective study, we analysed a large cohort of cancer patients with Gram-negative BSIs to identify key clinical and epidemiological data, along with their resistance profiles.

Prophylactic oral antibiotics are widely used in patients with high-risk hematologic malignancies. Patients with hematologic or oncologic malignancies are frequently hospitalized and exposed to broad-spectrum antibiotics, which can lead to alterations in their endogenous flora [5]. Understanding the changing epidemiology and resistance profile of BSIs with high mortality in cancer patients is critically important for promptly initiating appropriate antimicrobial therapy, which is vital for this patient group. A particularly alarming concern is the growing incidence of multidrug resistance among Gram-negative bacteria, which has become a major treatment challenge on a global scale [21–26].

Most studies investigating the bacterial profile and antibiotic susceptibility of BSIs in cancer patients have reported a predominance of Gram-negative bacteria [1, 3, 5, 7, 19–22, 26–28]. In a recent single-center study from Iran investigating the current epidemiology of BSI and its temporal changes, a predominance of Gram-negative was observed in cancer patients with BSIs [21]. Of the 414 positive blood cultures, Gram-negative bacteria was identified as the causative agent in 262 cases (63.3%). Rejthar et al*.* also reported higher rates of Gram-negative BSI (53%) than Gram-positive BSI (40%) in patients with hemato-oncological malignancy followed-up between January 2017 and September 2021 [26]. In the present study, episodes of Gram-negative bacteremia were observed predominantly in patients with newly diagnosed cancer (60%), which is consistent with previous studies reporting a prevalence of 70–80% in patients with patients without remission (*p* < 0.001)[1, 7]. The age and sex distributions are also consistent with the current literature, which shows a 60% male predominance and an age range of 47–62 years [7, 9, 10, 20–23].

The source of BSI has been reported in a limited number of studies involving cancer patients [3, 21, 29, 30]. Garcia-Vidal C et al*.* reported that catheters were the source of bacteremia in 35.8% of patients with acute leukaemia, whereas Chumbita M et al*.* reported that catheters were the cause of bacteremia in 21% of neutropenic patients with BSIs presenting with septic shock [3, 30]. Amanati et al*.* reported that CR-BSI accounted for 36.5% of positive blood cultures, while 63.5% were classified as primary BSI [21]. A catheter appears to be a significant source of Gram-negative BSIs, particularly in hematologic patients compared with those with solid organ malignancies. In the present study, catheters were the source of Gram-negative bacteremia in 11.1% of all episodes, with catheter-related bacteremia being significantly more common in patients with hematological malignancies (20.5%) than in those with oncological malignancies (1.1%) (*p* < 0.001). Marin M et al*.* reported similar findings, with catheters accounting for 25.4% of BSIs in patients with hematological malignancies and 7.0% in patients with solid organ malignancies, with a statistically significant difference (*p* < 0.001) [29]. Another study investigating bacteremia in solid organ patients supports these findings that the most common source of bacteremia was reported to be biliary origin (38.5%) [27].

Previous studies on Gram-negative BSIs in hemato-oncological patients consistently identified *E. coli*, *Klebsiella* spp., and *P. aeruginosa* as the three most frequently isolated microorganisms [3, 5, 19–22, 26]. In the present cohort, *E. coli* was the most frequently isolated Gram-negative bacteria, accounting for 40.6% of the cases, followed by *Klebsiella* spp. (28.8%) and *Pseudomonas* spp. (9.1%) in Gram-negative BSIs. Comparable findings have been reported in other studies. Rejthar et al*.* reported Gram-negative predominancy in BSIs, and *E. coli* (33%), *P. aeruginosa* (13%), and *Klebsiella* spp. (7%) were the most common pathogen in other than CR-BSI [27]. Similarly, in a study conducted by Choudhari et al*.* in India, *Escherichia coli* (20.3%) was reported as the most isolated pathogen among 687 isolates, followed by *Klebsiella* species (14.9%) and *Pseudomonas* species (14.8%) [28]. Several studies have identified *Klebsiella* spp. as the most frequently isolated Gram-negative pathogen [1, 7].

*E. coli* exhibited low susceptibility to third-generation cephalosporins (54.7% for ceftriaxone), quinolones (44.7% for ciprofloxacin), and trimethoprim/sulfamethoxazole (41.2%), with an ESBL rate of 40.3%. Quinolones and trimethoprim/sulfamethoxazole are commonly used in patients with hematological malignancies for both prophylaxis and treatment, and their use is widespread across all patient populations. The low susceptibility rates may be attributed to this frequent use. Similar results have been reported in several studies. Trecarichi et al. reported slightly lower susceptibility rates for *Enterobacteriaceae* isolates than did our study, with rates of 36.9% for third-generation cephalosporins and 20.1% for ciprofloxacin [20]. Several studies have investigated the prevalence of ESBL-producing *Enterobacteriaceae* in patients with hematological malignancies [21, 28, 31]. Data on the frequency of ESBL production have focused primarily on bloodstream isolates of *E. coli* and *K. pneumoniae*. In cancer patients, the prevalence of ESBL-producing isolates ranges from 12 to 75%, with an average of approximately 35% [21, 28, 31]. Amanati et al*.* reported an ESBL rate of 64.1% among *Enterobacteriaceae* strains causing BSI in adult patients with malignancies, which is notably higher than that reported in our study (40.3% for *E. coli* and 28.7% for *Klebsiella* spp.) in hemato-oncological cancer patients [21]. Similarly, Garcia-Vidal et al*.* reported an ESBL rate of 62.5% in *E. coli* strains isolated from patients with hematological malignancies, and Chen et al. reported a rate of 60.0% in a similar patient population. Choudhari et al*.* also reported a higher rate of ESBL producers in *E. coli* isolates (66%), and 36% in *Klebsiella* spp. isolates [28]. In a relatively small-sized study (77 episodes of Gram-negative BSIs) by Awada et al*.,* MDR pathogen rate was reported at 48% in patients with cancer [25]. These rates highlight the increasing prevalence of MDR organisms in vulnerable patient groups undergoing chemotherapy or HSCT, which poses significant challenges for empirical therapy due to the limited treatment options available [3, 7].

Piperacillin‒tazobactam is commonly used as a broad-spectrum empirical treatment option, particularly for managing infections in immunocompromised cancer patients. Its effectiveness against Gram-negative bacteria makes it a preferred choice for this high-risk patient population [4, 32]. However, resistance to piperacillin‒tazobactam is also increasing, particularly in nonfermenting Gram-negative bacteria. In our study, the susceptibility to piperacillin‒tazobactam was very low, even among *Enterobacterales* isolates (52.1%–67.7%). The susceptibility rates decreased to approximately 20% in nonfermenting Gram-negative bacteria, specifically 21.7% in *Pseudomonas* spp. and 33.3% in *Acinetobacter* spp. isolates. In the literature, there are varying rates of resistance to piperacillin‒tazobactam in cancer patients with BSIs [5, 7, 20, 31]. In a study of Trecarichi et al*.* from Italy, susceptibility rates were higher than those reported in our study: 69.8% among all Gram-negative BSIs, 83.4% among *E. coli,* 57.6% among *P. aeruginosa,* and 44.2% among *K. pneumoniae isolates*. Similarly, Carvalho et al*.* reported higher susceptibility rates (over 80%) in *E. coli*, *K. pneumoniae*, and *P. aeruginosa* isolates [5]. In contrast, a more recent study by Amanati et al*.* reported 100% resistance to piperacillin-tazobactam in all 123 *E. coli* isolates, which is consistent with our study results [21].

Carbapenems are critical antibiotics used to treat severe infections caused by MDR bacteria, especially in high-risk patients [4, 32]. However, resistance to these antibiotics among Gram-negative bacteria has become a critical concern, particularly in cancer patients with BSIs. In the present study, carbapenem resistance rates were found to be similar in *Klebsiella* spp. and *Pseudomonas* spp. isolates, both at approximately 27%, which is notably higher than the rate observed in *E. coli* isolates (9.1%), although a comparative statistical analysis was not performed. Several studies have reported varying rates of carbapenem resistance [5, 7, 19, 21, 25]. Current studies report high resistance rates to carbapenems, especially in nonfermenting Gram-negative bacteria. However, some studies have shown lower resistance rates. For example, Carvalho et al*.* reported meropenem resistance in 9.1% of *P. aeruginosa* isolates and 4.8% of *K. pneumoniae* isolates [5]. In another study, carbapenem resistance rate was noted in 9.1% of Gram-negative bacteremia episodes, although 48% of Gram-negative isolates was MDR pathogen [25]. In contrast, a multicenter study by Trecarichi et al. reported meropenem resistance in 20.8% of Gram-negative bacteria isolated from BSIs, with higher resistance rates in *P. aeruginosa* (71.2%) and *K. pneumoniae* (34.9%) although comparative statistical analysis was not performed, highlighting the challenges of treating these infections in immunocompromised patients with hematological malignancies [20]. Similarly, Amanati et al. reported high carbapenem resistance in nonfermenters (over 70%) and *Enterobacterales* (34%) [21]. Similar to our study, Rejthar et al*.* reported the rates of carbapenem resistance in 25% of *Pseudomonas* species and 53% of *Acinetobacter* species isolates [26].The increasing rates of carbapenem resistance in cancer patients are particularly alarming, as the timely initiation of effective antimicrobial therapy is critical for reducing mortality in this vulnerable group.

The emergence of antimicrobial resistance among Gram-negative bacteria in cancer patients is multifactorial and often driven by a combination of host-related and treatment-related factors. One of the most prominent contributors is the extensive and often prolonged use of broad-spectrum antibiotics, particularly in empiric regimens. Repeated or inappropriate antibiotic exposure exerts selective pressure on microbial populations, favoring the survival of resistant strains while eliminating susceptible ones [31, 33]. Profound and prolonged immunosuppression, neutropenia, frequent hospitalizations, previous colonization with resistant pathogens, the presence of central venous catheters, and intensive chemotherapy protocols create an ideal environment for subsequent infection by MDR organisms such as K*.* pneumoniae*,* P. aeruginosa, and A. baumannii*,* often limiting the efficacy of standard empiric therapies [31, 33, 34]. Therefore, incorporating up-to-date local resistance data and patient-specific risk factors into empirical therapy protocols is essential. Such a targeted approach not only enhances the likelihood of initiating effective therapy early, which is known to improve clinical outcomes, but also plays a critical role in antimicrobial stewardship by curbing unnecessary exposure to broad-spectrum agents [35]. Our data contribute to this growing body of evidence by providing current insights into local susceptibility patterns, which can support clinicians in optimizing empirical treatment strategies in high-risk oncology populations.

The strength of this study lies in the large number of patients and BSI episodes collected at a single center over a relatively short time period, along with the homogeneity of the data. Another strength of the study is the inclusion of both patient groups with hematological and solid organ malignancies at comparable rates.

We have some limitations in the study. First and foremost, we were not able to provide detailed empirical or targeted treatment regimens, as this is inherently a complex issue. In clinical practice, antibiotic therapies in these patients are frequently modified due to factors such as lack of clinical response or the ineffectiveness of empirical treatments against the isolated pathogens. Moreover, evaluating the administered therapies and treatment responses was beyond the scope of our study. Investigating the relationship between Gram-negative BSIs and clinical outcomes (mortality, length of the stay) and mortality risk factors would have been a valuable contribution to the literature. However, since the assessment of mortality involves various contributing factors, it was beyond the scope of this study and is currently being investigated in a separate ongoing project. The other limitation is that because the study was conducted in a single center, the findings may not be generalizable to other institutions or populations.

In conclusion, cancer patients, who are often immunocompromised due to chemotherapy or stem cell transplants, are at increased risk for infections caused by resistant pathogens. These infections are associated with increased morbidity and mortality due to limited treatment options and elevated resistance rates to commonly used antibiotics. The high prevalence of ESBL-producing and/or carbapenem-resistant MDR Gram-negative pathogens in patients with hematological malignancies, as demonstrated in the present study, underscores the importance of continuous monitoring of local epidemiological data and resistance profiles. This monitoring is essential for proper treatment management and the judicious use of antibiotics to treat infections effectively in this high-risk population.

## Data Availability

The datasets generated and/or analysed during the current study are available from the corresponding author upon reasonable request.

## References

[1] Kokkayil P, Agarwal R, Mohapatra S, Bakshi S, Das B, Sood S, et al. Bacterial profile and antibiogram of bloodstream infections in febrile neutropenic patients with haematological malignancies. J Infect Dev Ctries. 2018;12(6):442–7. 10.3855/jidc.9725. 31940295 10.3855/jidc.9725

[2] Blennow O, Ljungman P. The challenge of antibiotic resistance in haematology patients. Br J Haematol. 2016;172:497–511. 26492511 10.1111/bjh.13816

[3] Garcia-Vidal C, Cardozo-Espinola C, Puerta-Alcalde P, Marco F, Tellez A, Agüero D, et al. Risk factors for mortality in patients with acute leukemia and bloodstream infections in the era of multiresistance. PLoS ONE. 2018;13(6):e0199531. 10.1371/journal.pone.0199531. 29953464 10.1371/journal.pone.0199531 PMC6023133

[4] Freifeld AG, Bow EJ, Sepkowitz KA, Boeckh MJ, Ito JI, Mullen CA, et al. Clinical practice guideline for the use of antimicrobial agents in neutropenic patients with cancer: 2010 update by the Infectious Diseases Society of America. Clin Infect Dis. 2011;52(4):e56-93. 10.1093/cid/cir073. 21258094 10.1093/cid/cir073

[5] Carvalho AS, Lagana D, Catford J, Shaw D, Bak N. Bloodstream infections in neutropenic patients with haematological malignancies. Infect Dis Health. 2020;25(1):22–9. 31586572 10.1016/j.idh.2019.08.006

[6] Gudiol C, Bodro M, Simonetti A, Tubau F, González-Barca E, Cisnal M, et al. Changing aetiology, clinical features, antimicrobial resistance, and outcomes of bloodstream infection in neutropenic cancer patients. Clin Microbiol Infect. 2013;19(5):474–9. 22524597 10.1111/j.1469-0691.2012.03879.x

[7] Chen S, Zhang J, Liu X, Shi W, Shen Y. A practical update on the epidemiology and risk factors for the emergence and mortality of bloodstream infections from real-world data of 3014 hematological malignancy patients receiving chemotherapy. J Cancer. 2021;12(18):5494–505. 34405012 10.7150/jca.50802 PMC8364636

[8] Bow EJ. Infection in neutropenic patients with cancer. Crit Care Clin. 2013;29(3):411–41. 10.1016/j.ccc.2013.03.002. 23830647 10.1016/j.ccc.2013.03.002

[9] Martinez-Nadal G, Puerta-Alcalde P, Gudiol C, Calatayud L, Garcia-Vidal C, Cisnal M, et al. Inappropriate empirical antibiotic treatment in high-risk neutropenic patients with bacteremia in the era of multidrug resistance. Clin Infect Dis. 2019;70(6):1068–74. 10.1093/cid/ciz319 31321410

[10] Ayaz ÇM, Erdem H, Kula S, Altunbas Y, Şenol Y, Göker H. Factors associated with gram-negative bacteremia and mortality in neutropenic patients with hematologic malignancies in a high-resistance setting. Infect Dis Clin Microbiol. 2022;2:87–98. 10.36519/idcm.2022.141 PMC10985816 38633337

[11] Erdem H, Kula S, Senol Y, Gundogan K, Ibrahim F, Karaali, et al. Prospective analysis of febrile neutropenia patients with bacteremia: the results of an international ID-IRI study. Int J Antimicrob Agents. 2023;62(3):106919. 37423582 10.1016/j.ijantimicag.2023.106919

[12] Tang Y, Xiao H, Shen Y, Yang J, Yang J, Qiu H, et al. Prognostic factors and scoring model of haematological malignancies patients with bloodstream infections. Infection. 2018;46(4):513–21. 29767394 10.1007/s15010-018-1151-3

[13] Mermel LA, Allon M, Bouza E, Craven DE, Flynn P, O’Grady NP, et al. Clinical practice guidelines for the diagnosis and management of intravascular catheter-related infection: 2009 update by the Infectious Diseases Society of America. Clin Infect Dis. 2009;49(1):1–45. 19489710 10.1086/599376 PMC4039170

[14] Matuschek E, Åhman J, Webster C, Kahlmeter G. Antimicrobial susceptibility testing of colistin: evaluation of seven commercial MIC products against standard broth microdilution for *Escherichia coli*, *Klebsiella pneumoniae*, *Pseudomonas aeruginosa*, and *Acinetobacter* spp. Clin Microbiol Infect. 2018;24(8):865–70. 10.1016/j.cmi.2017.11.020. 29221995 10.1016/j.cmi.2017.11.020

[15] Han R, Shen S, Yin D, Ding L, Shi Q, Yang Y, et al. Performance of ceftazidime–avibactam 30/20-μg and 10/4-μg disks for susceptibility testing of Enterobacterales and Pseudomonas aeruginosa. Microbiol Spectr. 2023;11(2):e02720-e2722. 10.1128/spectrum.02720-22. 36744897 10.1128/spectrum.02720-22 PMC10100715

[16] European Committee on Antimicrobial Susceptibility Testing. Clinical breakpoints. https://www.eucast.org/clinical_breakpoints. Accessed 12 Aug 2025.

[17] Drieux L, Brossier F, Sougakoff W, Jarlier V. Phenotypic detection of extended-spectrum β-lactamase production in Enterobacteriaceae: review and bench guide. Clin Microbiol Infect. 2008;14:90–103. 18154532 10.1111/j.1469-0691.2007.01846.x

[18] Magiorakos AP, Srinivasan A, Carey RB, Carmeli Y, Falagas ME, Giske CG, et al. Multidrug-resistant, extensively drug-resistant and pandrug-resistant bacteria: an international expert proposal for interim standard definitions for acquired resistance. Clin Microbiol Infect. 2012;18(3):268–81. 21793988 10.1111/j.1469-0691.2011.03570.x

[19] Darakhshandeh A, Fathi E, Haji Gholami A, Ashrafi F, Mehrzad V, Nasri E. Bacterial spectrum and antimicrobial resistance pattern in cancer patients with febrile neutropenia. Int J Biochem Mol Biol. 2023;14(1):10–6. 36936611 PMC10018003

[20] Trecarichi EM, Pagano L, Candoni A, Pastore D, Cattaneo C, Fanci R, et al. Current epidemiology and antimicrobial resistance data for bacterial bloodstream infections in patients with hematologic malignancies: an Italian multicentre prospective survey. Clin Microbiol Infect. 2015;21(4):337–43. 25595706 10.1016/j.cmi.2014.11.022

[21] Amanati A, Ebadi M, Rezaei M, Ahmadi A, Azad M. Bloodstream infections in adult patients with malignancy, epidemiology, microbiology, and risk factors associated with mortality and multidrug resistance. BMC Infect Dis. 2021;21(1):636. 34215207 10.1186/s12879-021-06243-z PMC8254331

[22] Gedik H, Şimşek F, Kantürk A, Öğünç D. Bloodstream infections in patients with hematological malignancies: which is more fatal—cancer or resistant pathogens? Ther Clin Risk Manag. 2014;10:743–52. 25258539 10.2147/TCRM.S68450 PMC4172031

[23] Peri AM, Edwards F, Henden A, Harris PNA, Chatfield MD, Paterson DL, et al. Bloodstream infections in neutropenic and nonneutropenic patients with haematological malignancies: epidemiological trends and clinical outcomes in Queensland, Australia over the last 20 years. Clin Exp Med. 2023;23(8):4563–73. 37815735 10.1007/s10238-023-01206-x PMC10725384

[24] Gudiol C, Tubau F, Calatayud L, Garcia-Vidal C, Cisnal M, Sánchez-Ortega I, et al. Bacteraemia due to multidrug-resistant gram-negative bacilli in cancer patients: risk factors, antibiotic therapy and outcomes. J Antimicrob Chemother. 2011;66(3):657–63. 21193475 10.1093/jac/dkq494

[25] Awada B, Abarca J, Mumtaz S, Al-Khirbash A, Al-Sayegh H, Milupi M, et al. Predictors and outcomes of multi-drug-resistant gram-negative bacteremia in patients with cancer: a retrospective cohort study at a tertiary cancer center in Oman. IJID Regions. 2024;12:100399. 10.1016/j.ijregi.2024.100399. 39157419 10.1016/j.ijregi.2024.100399 PMC11326951

[26] Rejthar J, Desole M, Stroux A, Kremer P, Geerdts L, Kopf A, et al. Characteristics and antimicrobial therapy of bloodstream infections in tumour patients with special reference to antibiotic stewardship. J Cancer Res Clin Oncol. 2025;151(4):152. 10.1007/s00432-025-06204-y. 40289236 10.1007/s00432-025-06204-y PMC12034589

[27] de Dios-García B, Maestro G, Díaz-Pedroche C, Parra W, Campos Ó, Orellana MÁ, et al. Bacteremia in patients with solid organ cancer: insights into epidemiology and antibiotic consumption. Cancers (Basel). 2023;15(23):5561. 10.3390/cancers15235561. 38067264 10.3390/cancers15235561 PMC10705097

[28] Choudhari S, Gawande R, Watchmaker J, Bamnote P, Mishra P, Dwivedi P. Bloodstream infections in cancer patients in central India: pathogens and trends of antimicrobial resistance over a 5-year period. Access Microbiol. 2024;6(10):000673.v5. 10.1099/acmi.0.000673.v5. 39474270 10.1099/acmi.0.000673.v5 PMC11521250

[29] Marin M, Gudiol C, Ardanuy C, Garcia-Vidal C, Calvo M, Arnan M, et al. Bloodstream infections in neutropenic patients with cancer: differences between patients with haematological malignancies and solid tumours. J Infect. 2014;69(5):417–23. 24960295 10.1016/j.jinf.2014.05.018

[30] Chumbita M, Puerta-Alcalde P, Gudiol C, Garcia-Pouton N, Laporte-Amargós J, Ladino A, et al. Impact of empirical antibiotic regimens on mortality in neutropenic patients with bloodstream infection presenting with septic shock. Antimicrob Agents Chemother. 2022;66(2):e0174421. 10.1128/aac.01744-21. 34843387 10.1128/AAC.01744-21 PMC8846468

[31] Trecarichi EM, Tumbarello M. Antimicrobial-resistant gram-negative bacteria in febrile neutropenic patients with cancer: current epidemiology and clinical impact. Curr Opin Infect Dis. 2014;27(2):200–10. 10.1097/QCO.0000000000000065. 24573013 10.1097/QCO.0000000000000038

[32] Averbuch D, Orasch C, Cordonnier C, Livermore DM, Mikulska M, Viscoli C, et al. European guidelines for empirical antibacterial therapy for febrile neutropenic patients in the era of growing resistance: summary of the 2011 4th European Conference on Infections in Leukemia. Haematologica. 2013;98(12):1826–35. 10.3324/haematol.2013.091025. 24323983 10.3324/haematol.2013.091025 PMC3856957

[33] Tamma PD, Aitken SL, Bonomo RA, Mathers AJ, van Duin D, Clancy CJ. Infectious diseases society of america guidance on the treatment of antimicrobial-resistant gram-negative infections. Clin Infect Dis. 2021;72(7):e169–83. 33106864 10.1093/cid/ciaa1478

[34] Gudiol C, Albasanz-Puig A, Laporte-Amargós J, Pallarès N, Mussetti A, Ruiz-Camps I, et al. Clinical predictive model of multidrug resistance in neutropenic cancer patients with bloodstream infection due to Pseudomonas aeruginosa. Antimicrob Agents Chemother. 2020;64(3):e02494-e2519. 32015035 10.1128/AAC.02494-19 PMC7179320

[35] Gudiol C, Aguado JM, Carratalà J. Bloodstream infections in patients with solid tumors. Virulence. 2019;7(3):298–308. 10.1080/21505594.2016.1141161 PMC4871686 26787095

